# Endothelial Cell-Selective Adhesion Molecule Expression in Hematopoietic Stem/Progenitor Cells Is Essential for Erythropoiesis Recovery after Bone Marrow Injury

**DOI:** 10.1371/journal.pone.0154189

**Published:** 2016-04-25

**Authors:** Takao Sudo, Takafumi Yokota, Daisuke Okuzaki, Tomoaki Ueda, Michiko Ichii, Tomohiko Ishibashi, Tomomi Isono, Yoko Habuchi, Kenji Oritani, Yuzuru Kanakura

**Affiliations:** 1 Department of Hematology and Oncology, Osaka University Graduate School of Medicine, Suita, Osaka, Japan; 2 DNA Chip Development Center, Research Institute for Microbial Diseases, Osaka University, Suita, Osaka, Japan; B.C. Cancer Agency, CANADA

## Abstract

Numerous red blood cells are generated every second from proliferative progenitor cells under a homeostatic state. Increased erythropoietic activity is required after myelo-suppression as a result of chemo-radio therapies. Our previous study revealed that the endothelial cell-selective adhesion molecule (ESAM), an authentic hematopoietic stem cell marker, plays essential roles in stress-induced hematopoiesis. To determine the physiological importance of ESAM in erythroid recovery, ESAM-knockout (KO) mice were treated with the anti-cancer drug, 5-fluorouracil (5-FU). ESAM-KO mice experienced severe and prolonged anemia after 5-FU treatment compared to wild-type (WT) mice. Eight days after the 5-FU injection, compared to WT mice, ESAM-KO mice showed reduced numbers of erythroid progenitors in bone marrow (BM) and spleen, and reticulocytes in peripheral blood. Megakaryocyte-erythrocyte progenitors (MEPs) from the BM of 5-FU-treated ESAM-KO mice showed reduced burst forming unit-erythrocyte (BFU-E) capacities than those from WT mice. BM transplantation revealed that hematopoietic stem/progenitor cells from ESAM-KO donors were more sensitive to 5-FU treatment than that from WT donors in the WT host mice. However, hematopoietic cells from WT donors transplanted into ESAM-KO host mice could normally reconstitute the erythroid lineage after a BM injury. These results suggested that ESAM expression in hematopoietic cells, but not environmental cells, is critical for hematopoietic recovery. We also found that 5-FU treatment induces the up-regulation of ESAM in primitive erythroid progenitors and macrophages that do not express ESAM under homeostatic conditions. The phenotypic change seen in macrophages might be functionally involved in the interaction between erythroid progenitors and their niche components during stress-induced acute erythropoiesis. Microarray analyses of primitive erythroid progenitors from 5-FU-treated WT and ESAM-KO mice revealed that various signaling pathways, including the GATA1 system, were impaired in ESAM-KO mice. Thus, our data demonstrate that ESAM expression in hematopoietic progenitors is essential for erythroid recovery after a BM injury.

## Introduction

Numerous hematopoietic cells are perpetually generated from hematopoietic stem/progenitor cells that exist primarily in the bone marrow (BM) after birth. Among the various types of hematopoietic cells, red blood cells are indispensable to maintain our day-to-day activities throughout life. Indeed, 2–3 million erythrocytes are produced each second in the adult human. An increase in erythropoiesis is required in times of stress, particularly after receiving chemo-radio therapy for cancer treatment.

It is not hematopoietic stem cells (HSCs) or multi-potent hematopoietic progenitor cells (HPCs), but erythroid-specific highly proliferative progenitors, that are thought to play critical roles in supporting the large daily output of red blood cells. Progenitors at the burst forming unit-erythrocyte (BFU-E) level are likely to constitute immature erythroid-restricted progenitors, which possess considerable proliferation potential [1]. These progenitors progressively differentiate into erythroblasts and reticulocytes to produce a tremendous number of mature erythrocytes. Additionally, macrophages appear to play important roles during differentiation. A structural unit called the erythroblastic island, which consists of a central macrophage surrounded by erythroid progenitors at various differentiation stages, can be found in the fetal liver and the BM [2]. Furthermore, Chow et al. have recently shown that CD169^+^ macrophages promote erythroid maturation under both homeostatic and stress conditions by acting like a “niche” for erythroblasts [3].

Accumulating evidence has indicated that molecular crosstalk between erythroblasts and macrophages is important for late erythrocyte maturation. Cell surface proteins such as integrin families or adhesion molecules have been shown to mediate interactions between erythroblasts and central macrophages [4, 5]. Soni et al. reported that the erythroblast macrophage protein, which is expressed in both erythroblasts and macrophages, mediates cell-cell interactions and is required for erythroblast enucleation [6]. However, numerous questions regarding the molecular mechanisms mediating the interactions between early erythroid progenitor cells and their environment remain to be answered.

We previously reported that endothelial cell-selective adhesion molecule (ESAM) expression is a biomarker of HSCs in mice and is useful to trace the activation of HSCs upon BM injury [7, 8]. ESAM is functionally important for hematopoiesis because ESAM deficiency causes severe BM suppression after administration of the anti-cancer drug, 5-fluorouracil (5-FU) [7]. Among the diverse hematopoietic lineages, the erythroid lineage is the most sensitive to ESAM deficiency. However, it remains unclear what stage of erythropoiesis is impaired and what mechanisms are involved in the severe and prolonged anemia seen in ESAM-deficient mice after 5-FU treatment. In this study, we analyzed how ESAM deficiency influences the early stages of erythropoiesis. Our data provide a novel scenario in which ESAM expression in hematopoietic progenitors plays a critical role in restoring erythropoiesis after BM injury.

## Materials and Methods

### Mice

Wild-type (WT) C57BL/6 mice were obtained from CLEA Japan (Shizuoka, Japan). ESAM-knockout (KO) mice were developed by Dr. T. Ishida (Kobe University, Japan) [9]. Mating of heterozygous male and female mice was routinely performed to generate homozygous and heterozygous ESAM-KO and WT mice. Three types of PCR primers were used to genotype ESAM-KO mice, as previously documented [10]. The congenic C57BL/6 strain (C57BL/6SJL; CD45.1 alloantigen) was purchased from Jackson Laboratories (Bar Harbor, ME) and used for transplantation experiments. All mice used in this study were 8 to 12 weeks old. Animal studies were performed with the approval of the Institutional Review Board of Osaka University (Permit Number: 25-098-006).

### Antibodies and Reagents

The 5-FU was purchased from Kyowa-Hakko Kirin (Tokyo, Japan). Purified anti-Ly6G and Ly6C/Gr1 (RB6-8C5) monoclonal antibodies (mAbs), phycoerythrin (PE)-conjugated anti-CD3e (145-2C11), Mac1 (M1/70), Ly6G and Ly6C/Gr1 (RB6-8C5), Ter119, CD45.1 (A20), and CD71 (C2) mAbs, fluorescein isothiocyanate (FITC)-conjugated anti-CD34 (RAM34), CD11b/Mac1 (M1/70), Ly6G and Ly6C/Gr1 (RB6-8C5), CD45R/B220 (RA3-6B2), Ter119, and CD3e (145-2C11) mAbs, allophycocyanin (APC)-conjugated anti-CD117/c-Kit (2B8) mAb, phycoerythrin-Cy7 (PE-Cy7)-conjugated anti-Sca1 (Ly6A/E; D7) mAb, PerCPCy5.5-conjugated anti-CD45.2 (104) mAb, Alexa Fluor 647-conjugated anti-CD19 (1D3) mAb, streptavidin- phycoerythrin (SAv-PE), and streptavidin- phycoerythrin-Texas red (SAv-PE-TR) were purchased from BD Pharmingen (San Diego, CA). Purified anti-CD3 (17A2), Mac1 (M1/70), and Ter119 mAbs, PE-conjugated anti-CD105/Endoglin (MJ7/18) and CD115 (AFS98) mAbs, PE-Cy7-conjugated anti-Ly6G and Ly6C/Gr1 (RB6-8C5) mAb, biotinylated anti-CD41 (eBioMWReg30) mAb, and PerCPCy5.5-conjugated anti-CD16/CD32 (93) mAb were purchased from eBioscience (San Diego, CA). PE-conjugated anti-CD150 (TC15-12F12.2), CD45R/B220 (RA3-6B2), and IL7Rα (A7R34) mAbs, FITC-conjugated anti-F4/80 (BM8) mAb, APC-Cy7-conjugated anti- CD16/CD32 (93) mAb, and PerCPCy5.5-conjugated anti- CD117/c-Kit (2B8) mAb were purchased from BioLegend (San Diego, CA). A rat anti-mouse ESAM (1G8) mAb was purchased from BioLegend. The antibody was biotinylated in our laboratory using Sulfo-NHS-LC-Biotin (Thermo Fisher Scientific, Rockford, IL).

### Flow Cytometry

Cells were obtained from adult mouse tissues as indicated in each experiment. Splenocytes and BM cells of the femora and tibiae were resuspended in staining buffer (PBS containing 3% fetal bovine serum). Then, the cells were stained with the indicated antibodies. Lin Abs contained anti-Mac1, Gr1, CD3e, CD45R/B220, and Ter119 Abs. A biotinylated anti-ESAM Ab was developed with SAv-PE or SAv-PE-TR. PE-conjugated, SAv-PE, and SAv-PETR Abs were diluted 1:100, and other Abs were diluted 1:50 in staining buffer. Flow cytometry analyses were performed with FACSAria or FACSCanto (BD Bioscience, San Jose, CA). Data analyses were performed using the FlowJo software (Tree Star, San Carlos, CA). Absolute cell numbers in each population from a pair of femora and tibiae, or spleen, was calculated by multiplying total cell count by its percentage.

### Detection of Erythroblastic Islands

The experiments were performed following a protocol described by Chow et al. [3]. Briefly, flushed BM cells were stained with F4/80-FITC and Ter119-PE Abs for 2 h at room temperature. Then, cells were diluted 3.5-fold in FACS buffer and flow cytometry analyses were performed using an SH800 cell sorter (Sony, Tokyo, Japan).

### Cell Isolation

WT and ESAM-KO mice were treated with a single 5-FU injection (120 mg/kg) and sacrificed 8 days after treatment. BM cells obtained from the femora and tibiae were stained with FITC- anti-Lin Abs in combination with PE-anti-CD150, PerCPCy5.5-anti-c-Kit, APC-anti-endoglin, PE-Cy7-anti-Sca1, APC-Cy7-anti-FCγR, and biotinylated anti-CD41 Abs. Subsequent to staining with SAv-PE-TR, Lin^-^ FCγR^-/Lo^ CD41^Lo^ c-Kit^+^ Sca1^-^ endoglin^+^ CD150^+^ pre colony forming unit-erythrocyte (CFU-E) cells were sorted with FACSAria (BD Bioscience). The purity of the sorted cells was routinely confirmed by using a portion of each sorted population, and was determined to be higher than 97%.

### Methylcellulose Cultures

Splenocytes and BM cells were isolated from WT and ESAM-KO mice. Nucleated cells were plated in methylcellulose media (StemCell Technologies, Vancouver, Canada). Then, 2 × 10^5^ or 1 × 10^5^ BM cells were plated for counting BFU-E or CFU-E, respectively. At 5-FU day 0, 2 × 10^5^ splenocytes were plated to count both BFU-E and CFU-E. 1 × 10^6^ or 2 × 10^5^ 5-FU-treated splenocytes (day 8) were plated for counting BFU-E or CFU-E, respectively. Cells were plated in Methocult M3334 for CFU-E assays and in Methocult SF M3436 for BFU-E assays. To evaluate colony numbers in peripheral blood (PB), nucleated cells derived from 100 μL of PB were plated in methylcellulose media (Methocult GF M3434). To evaluate colony numbers in sorted common myeloid progenitors (CMPs) or granulocyte-monocyte progenitors (GMPs), cells were plated in Methocult GF M3434. CFU-E and BFU-E were scored at 3 and 9 days after incubation, respectively. Granulocyte colony-forming units (CFU-G), macrophage colony-forming units (CFU-M), granulocyte-macrophage colony-forming units (CFU-GM), and mixed erythroid-myeloid colony-forming units (CFU-Mix) were scored at 8 to 10 days after incubation. An absolute number of each colony isolated from a pair of femora and tibiae or spleen was calculated by colony count and total cell number.

### Microarray

Total RNA samples from sorted cells were isolated using the RNeasy Mini Kit (Qiagen, Venlo, Netherlands). Then, the RNA samples were amplified and converted into cDNA using the ovation Pico WTA System V2 (NuGEN, San Carlos, CA). Gene expression profiling using the SurePrint G3 mouse 8x60K microarray assay (Agilent Technologies, Santa Clara, CA) was performed by Takara Bio (Otsu, Japan). Two criteria were used to normalize and compare the values of WT and ESAM-KO pre CFU-E and define the signal ratios: more than a 2-fold increase or less than a 2-fold decrease. Bioinformatic analyses were conducted with Ingenuity Pathway Analysis software (Ingenuity Systems; Qiagen). The raw data have been accepted in Gene Expression Omnibus (GEO), a public repository for microarray data, aimed at storing Minimum Information About Microarray Experiments (MIAME). Access to data concerning this study can be found under GEO experiment accession number (GSE 73496).

### Statistical Methods

Statistical analyses were conducted using a standard Student’s *t*-test. Error bars used throughout represent the standard error of the mean (SEM). Statistical significance was taken at values of *P < 0.05, **P < 0.01, and ***P < 0.001.

## Results

### ESAM Deficiency Causes Severe Anemia after 5-FU Treatment

**Fig 1:**
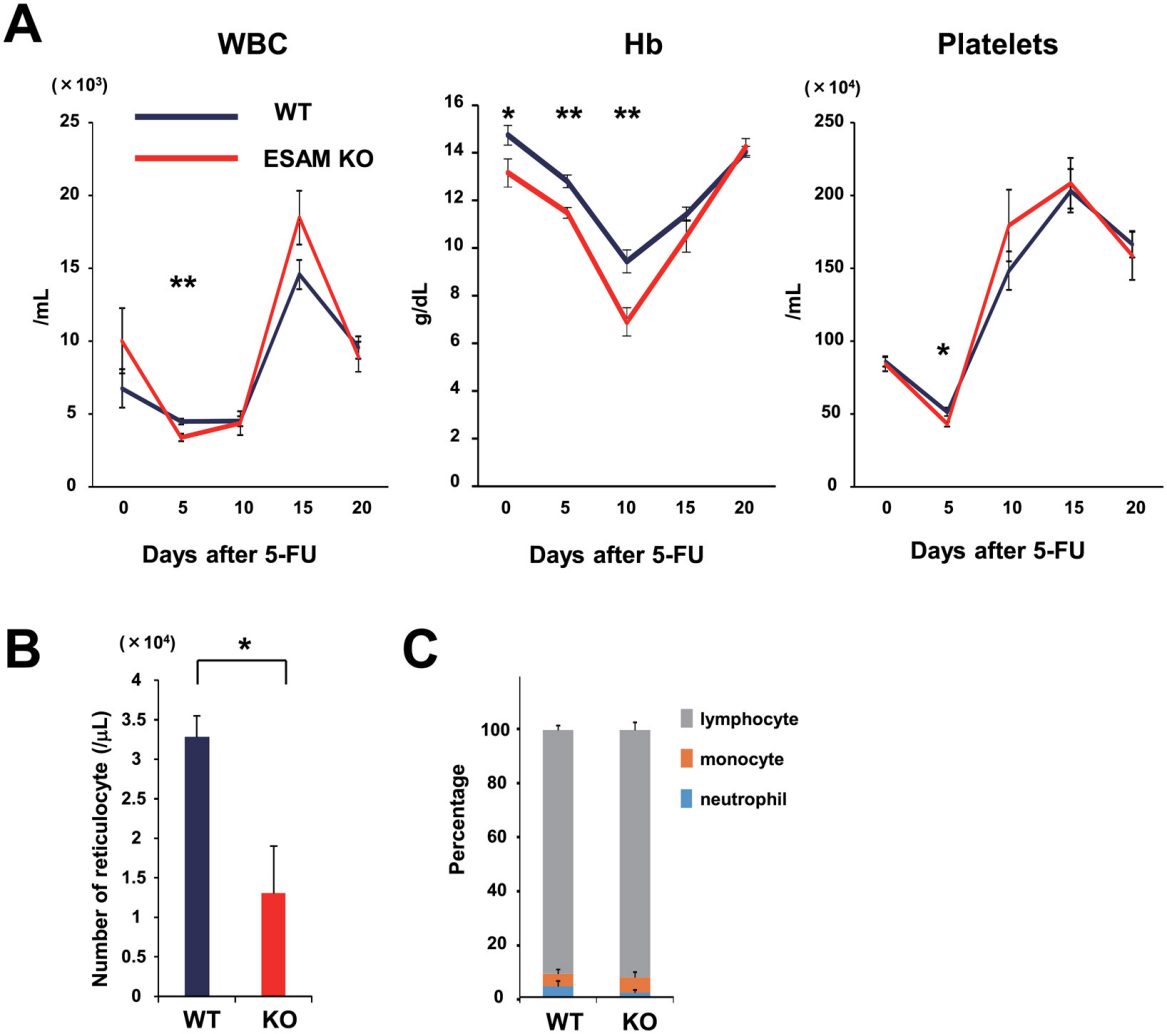
ESAM deficiency caused serious and prolonged anemia after 5-FU treatment. (A) WT or ESAM-KO mice were intravenously administered with a single 200 mg/kg dose of 5-FU to then examine the PB every 5 days (up to day 20) using a blood cell analyzer (KX-21, Sysmex) (n = 10 in each). WBC, Hb, and platelet counts were plotted. Two ESAM-KO mice died between day 10 and day 15 after 5-FU treatment. (B, C) WT or ESAM-KO mice were administered 150 mg/kg of 5-FU and PB analyses were performed at day 8. (B) The number of reticulocytes were quantified by visual counting (n = 3). (C) The mean percentage of neutrophil, monocyte, and lymphocyte subsets in WBC were quantified by visual counting (n = 3). Data are shown as mean ± SEM. Statistically significant differences are represented by asterisks (*P < 0.05, ** *P* < 0.01).

We have previously reported that ESAM-KO mice exhibit a more life-threatening pancytopenia than WT mice within 10 days after a single 5-FU injection [7]. In this study, we administered 5-FU to WT and ESAM-KO mice and monitored blood cell counts up to day 20 (Fig 1A). Once again, we confirmed that ESAM deficiency caused severe pancytopenia. Twenty percent of ESAM-KO mice showed a severe reduction in both the white blood cell (WBC) count and hemoglobin (Hb) levels, and died before hematopoietic recovery. The surviving ESAM-KO mice sharply recovered their WBC count and platelet levels after day 10, but their anemia was severe and prolonged in comparison to WT mice. Indeed, in more than half of the 5-FU-treated ESAM-KO mice, the Hb values fell under 7 g/dL on day 10. The reticulocyte number in the PB of 5-FU-treated ESAM-KO mice at day 8 was significantly reduced compared to that of WT mice (Fig 1B). Ooi et al. reported that ESAM-KO mice exhibited no differences in the frequencies of mature cell populations [11]. WBC subset analysis showed that 5-FU-treated ESAM-KO mice also exhibited no differences in the percentage of neutrophil, monocyte, and lymphocyte subsets (Fig 1C). These results suggested that erythropoiesis dysfunction, particularly after BM injury, is a prominent phenotype in ESAM-KO mice.

### ESAM Deficiency Reduces Erythroid Progenitors in Spleen, but Not in BM, under Homeostatic Conditions

**Fig 2:**
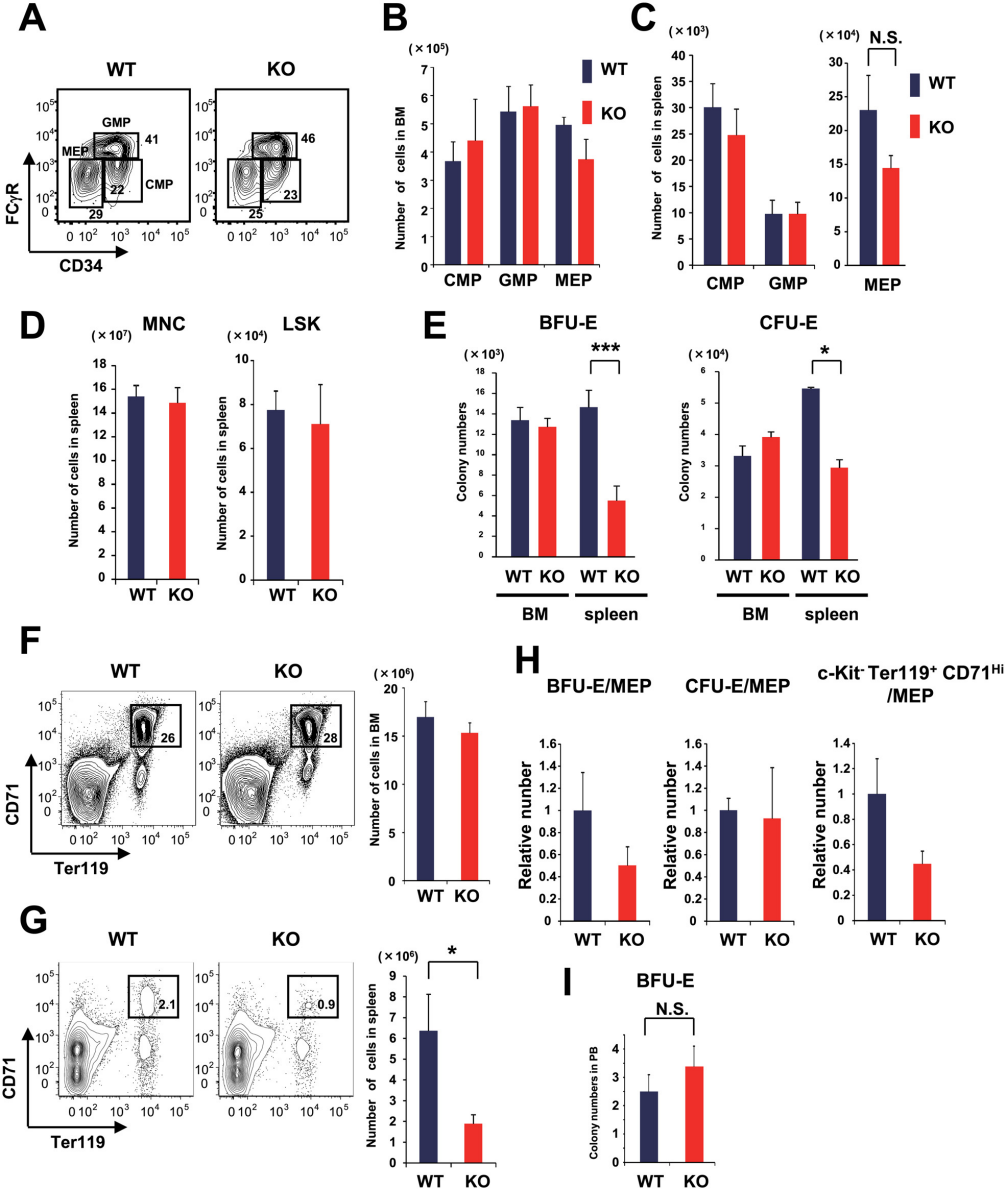
ESAM deficiency caused reduced erythropoiesis potential in the spleen, but not in the BM. WT and ESAM-KO mice were sacrificed, and flow cytometry (FACS) analyses or methylcellulose colony cultures were performed. (A) The representative FACS profiles of the Lin^-^ IL-7Rα^-^ c-Kit^+^ Sca1^-^ fraction of WT and ESAM-KO BM cells are shown. Each number indicates the percentage of FCγR^Lo^ CD34^+^ CMP, FCγR^Hi^ CD34^+^ GMP, or FCγR^Lo^ CD34^-^ MEP population within the Lin^-^ IL-7Rα^-^ c-Kit^+^ Sca1^-^ fraction. (B) The numbers of CMPs, GMPs, and MEPs in the BM are shown (n = 5 in each). (C) The numbers of CMPs, GMPs, and MEPs in the spleen are shown (n = 4 in each). (D) The number of total mononuclear cells (MNC) and LSK cells in the spleen are shown (n = 4 in each). (E) BM cells and splenocytes were subjected to methylcellulose colony formation assays for counting BFU-E and CFU-E. Each bar represents the number of BFU-E (left panel) or CFU-E (right panel) in the BM and spleen (BM; n = 6 in each, spleen; n = 5 in each). (F-G) Representative FACS profiles of c-kit^-^ fractions of BM cells (F) and splenocytes (G) from WT and ESAM-KO mice are shown. In the panels, each number indicates the percentage of the Ter119^+^ CD71^Hi^ population in the c-Kit^-^ fraction. The numbers of c-Kit^-^ Ter119^+^ CD71^Hi^ cells in the BM and spleen are shown in the right graphs (n = 5 in each). (H) The relative numbers of BFU-E, CFU-E, and c-Kit^-^ Ter119^+^ CD71^Hi^ progenitors divided by the number of MEP in WT or ESAM-KO mice’s spleen are shown. (I) The number of BUF-E in nucleated cells derived from 100 μL of PB is shown (n = 4 in each). The blue bars represent the results for the WT mice, and the red bars represent those of ESAM-KO mice. Data are from one of two independent experiments that gave similar results. Data are shown as mean ± SEM. Statistically significant differences are represented by asterisks (*P < 0.05, **P < 0.01, *** *P* < 0.001).

ESAM-KO mice showed slight anemia, even under homeostatic conditions. Because the number of Lin^-^ Sca-1^+^ c-Kit^Hi^ (LSK) cells in the BM, which contains HSCs and multipotent HPCs, did not differ between WT and ESAM-KO mice [7, 12], we inferred that additional lineage-restricted progenitors might be affected, resulting in the anemic phenotype observed in ESAM-KO mice. Thus, we first examined the Lin^-^ Sca-1^-^ c-Kit^+^ fraction, which contains non-lymphoid multipotent progenitors, and estimated the number of common myeloid progenitors (CMPs), granulocyte-monocyte progenitors (GMPs), and megakaryocyte-erythrocyte progenitors (MEPs) [12]. We found no significant differences between the homeostatic BM progenitors of WT and ESAM-KO mice (Fig 2A and 2B). Methylcellulose colony assays for BFU-E and CFU-E showed no differences between WT and ESAM-KO mice (Fig 2E). Flow cytometry of cells stained with anti-c-Kit, Ter119, and CD71 antibodies could identify the later stage of erythroid progenitors as c-Kit^-^ Ter119^+^ CD71^Hi^ or erythroblast subpopulations defined by forward scatter (FSC) in combination with Ter119 and CD71 [13, 14]. There was no obvious reduction, even in this mature erythroid progenitor population of ESAM-KO mice (Fig 2F and S1A and S1B Fig). These results suggested that ESAM deficiency does not affect erythropoiesis in the BM under homeostatic conditions.

A comparative evaluation was also performed for the spleen. While no apparent differences were observed in the number of total mononuclear cells (MNC), LSK cells, CMPs, and GMPs between WT and ESAM-KO mice, a slight decrease in MEPs in ESAM-KO mice’s spleens was repeatedly detected (Fig 2C and 2D). A significant reduction was observed in colony formation of both BFU-E and CFU-E from ESAM-KO mice’s spleens (Fig 2E). Furthermore, proerythroblasts, c-Kit^-^ Ter119^+^ CD71^Hi^, or Ter119^Hi^ CD71^Hi^ FSC^Lo^ mature erythroid progenitor levels also significantly decreased (Fig 2G and S1C and S1D Fig). To compare erythroid maturation from MEP levels in the spleen, we determined counts of each progenitor per MEP. A reduction of BFU-E and c-Kit^-^ Ter119^+^ CD71^Hi^ counts (calculated per MEP) was observed in ESAM-KO mice compared to that in WT mice, but this difference was not significant (Fig 2H). Because BFU-E counts from PB showed no differences between WT and ESAM-KO mice, circulating erythroid progenitors were not increased in ESAM-KO mice at steady state (Fig 2I). Taken together, these results suggest that erythroid cell production, particularly in the spleen, is compromised in ESAM-KO mice. This might account for the slight anemic phenotype of steady state ESAM-KO mice [7].

### Erythropoiesis Was Severely Damaged in ESAM-KO Mice after 5-FU Treatment

**Fig 3:**
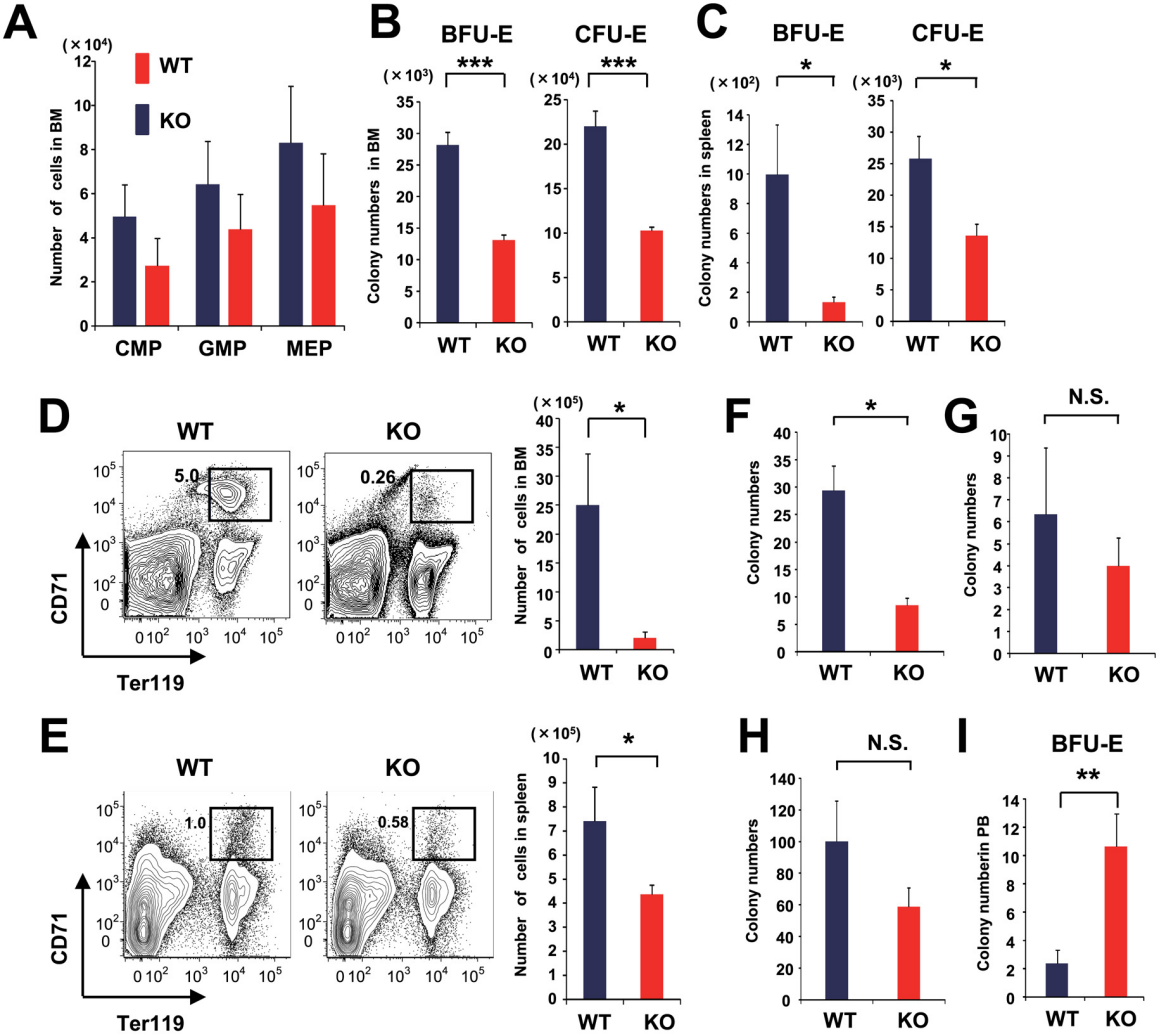
Erythroid progenitors in the BM and spleen are reduced in 5-FU-treated ESAM-KO mice. (A-I) WT and ESAM-KO mice were treated with 150 mg/kg of 5-FU, and FACS analyses or methylcellulose cultures were performed at day 8. Then, the number of myeloid and erythroid progenitors from BM, spleen, or PB was evaluated. (A) The number of CMPs, GMPs, and MEPs in BM is shown (n = 4 in each). (B-C) BM cells and splenocytes were subjected to methylcellulose colony formation assays for counting BFU-E and CFU-E. Each bar represents the number of BFU-E (left panel) or CFU-E (right panel) in BM (B) and spleen (C) (n = 6 in each). (D-E) In the left panels, representative FACS profiles of BM (D) and spleen (E). In the right panels, quantification of c-Kit^-^ Ter119^+^ CD71^Hi^ cells in BM (D) and spleen (E) (BM; n = 7 in each, spleen; n = 6 in each). (F-H) CMPs, GMPs, and MEPs were sorted from 5-FU-treated BM of WT or ESAM-KO mice. Then, 1 × 10^3^ MEPs, 2× 10^2^ CMPs, or 1 × 10^3^ GMPs were subjected to methylcellulose colony formation assays for counting BFU-E (F), CFU-Mix (G), or CFU-G/M/GM (H), respectively (n = 3 in each). (I) The numbers of BUF-E in nucleated cells derived from 100 μL of PB are shown (n = 5 in each). (A-I) The blue bars represent the results for the WT mice, and the red bars represent those of ESAM-KO mice. All data are from one of two independent experiments that gave similar results. Data are shown as mean ± SEM. Statistically significant differences are represented by asterisks (*P < 0.05, ** *P* < 0.01, *** *P* < 0.001).

Erythropoiesis in ESAM-KO mice was found to be sensitive to 5-FU treatment (Fig 1A). To determine the stage at which the erythroid lineage differentiation was sensitive to the myelo-suppressive treatment, WT and ESAM-KO mice were treated with 150 mg/kg of 5-FU to perform a detailed evaluation of the erythroid progenitors in the BM and the spleen, 8 days after treatment. We observed that CMPs, GMPs, and MEPs were decreased in the BM of ESAM-KO mice, although these differences were not statistically significant due to a wide deviation among mice (Fig 3A). Colony assays showed that BM cells from ESAM-KO mice contained significantly fewer BFU-E and CFU-E (Fig 3B). Additionally, the number of c-Kit^-^ Ter119^+^ CD71^Hi^ mature erythroid progenitors in the BM of ESAM-KO mice was drastically reduced compared to that of WT mice (Fig 3D). The proerythroblast (Ter119^Med^ CD71^Hi^) population in BM cells and CD71^Hi^ FSC^Hi^ or the CD71^Hi^ FSC^Lo^ erythroblast population in Ter119^Hi^ cells was smaller in ESAM-KO mice compared to that in WT mice (S2A and S2B Fig). Next, to assess the colony-forming activity of each myeloid progenitor, we sorted CMPs, GMPs, and MEPs from 5-FU-treated BM, and performed colony formation assays. While MEPs, CMPs, or GMPs from ESAM-KO mice showed lower BFU-E, CFU-Mix, or CFU-G/M/GM forming activities, respectively, only the reduction of BFU-E derived from ESAM-KO MEPs was drastic (71% reduction) and significant compared to WT mice (Fig 3F–3H). These results suggested that ESAM expression is indispensable for erythroid proliferation from MEPs after 5-FU treatment.

Because the spleen serves as the main tissue for erythropoiesis after acute anemia [15–17], we also analyzed erythroid progenitors in the spleen after the 5-FU treatment. We found that splenocytes of ESAM-KO mice had lower colony forming activities of both BFU-E and CFU-E, although the total numbers of splenocytes in ESAM-KO mice were not significantly reduced compared to WT mice (Fig 3C and data not shown). We also detected a severe reduction of c-Kit^-^ Ter119^+^ CD71^Hi^ erythroid progenitors in ESAM-KO mice’s spleens (Fig 3E). FACS profiles of splenocytes with respect to erythroblast subpopulations are shown in S2C and S2D Fig. BFU-E counts from PB in 5-FU-treated ESAM-KO mice were significantly higher than WT mice (Fig 3I). It is possible that the reduced homing capacity of erythroid progenitors in ESAM-KO mice enhances the anemic phenotype after BM injury.

We also assessed the erythropoiesis response in a hemolytic anemia model induced by phenyl hydrazine (PHZ) treatment. Slight anemia was observed in ESAM-KO mice after PHZ administration in a trend consistent with that seen in the homeostatic condition (S3A Fig). However, we did not observe any significant differences between WT and ESAM-KO mice in the number of BFU-E or c-Kit^-^ Ter119^+^ CD71^Hi^ erythroid progenitors in the BM or spleen (S3B and S3C Fig). These results suggested that ESAM is not essential for erythroid proliferation after a red blood cell loss condition.

Next, we examined if hemolysis or defective production of erythropoiesis-related molecules might be involved in the severe anemia seen in ESAM-KO mice after 5-FU treatment. Serum bilirubin levels of 5-FU-treated ESAM-KO at day 8 were not significantly higher (compared to WT mice) (S4A Fig). Serum erythropoietin (EPO) levels of WT and ESAM-KO mice did not differ under homeostatic conditions (data not shown), and EPO concentrations in ESAM-KO mice sera increased after 5-FU treatment to levels higher than those in WT sera (S4B Fig). In addition, mRNA expression level of the erythropoietin receptor (*EpoR*) in ESAM-KO immature erythroid progenitors was higher compared to that in WT mice (S4C Fig). Regarding stem cell factor (SCF) expression, both protein and mRNA levels in the BM and the spleen were similar between 5-FU-treated WT and ESAM-KO mice (S4D and S4E Fig). Bone morphogenetic protein-4 (BMP-4) is also a stimulatory factor of stress erythropoiesis, especially in the recovery of acute anemia [17, 18]. The mRNA expression levels of *Bmp-4* in the BM and the spleen of 5-FU-treated ESAM-KO mice were comparable with those of 5-FU-treated WT mice at day 8 (S4F Fig). These results suggested that ESAM plays important roles in promoting acute erythropoiesis in both BM and spleen after myelo-suppressive events. The sensitivity of erythropoiesis in ESAM-KO mice is likely due to ESAM deficiency and not by indirect mechanisms including hemolysis or insufficient production of EPO, SCF, and BMP-4.

### ESAM Expression in Hematopoietic Cells Is Necessary for Hematopoietic Recovery

**Fig 4:**
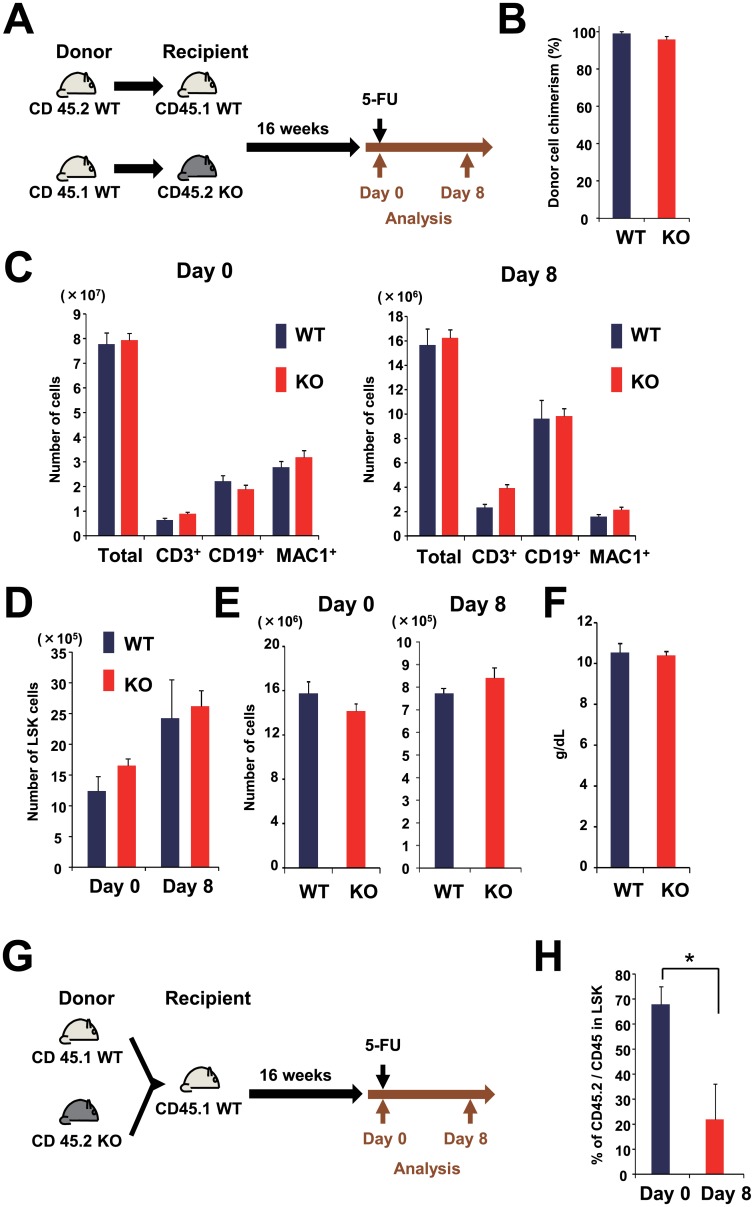
ESAM expression in hematopoietic cells is required for hematopoietic recovery after BM injury. (A-F) 1 × 10^6^ BM cells from CD45.1 WT or CD45.2 WT mice were transplanted to lethally irradiated CD45.2 ESAM-KO or CD45.1 WT mice, respectively (n = 8 in each group). Sixteen weeks after transplantation, half of each group was sacrificed at 5-FU day 0, and others were treated with 150 mg/kg of 5-FU and sacrificed at day 8. (A) A scheme of the transplantation protocol is shown. (B) PB chimerisms that were analyzed by FACS are shown. The blue bar represents the percentages of CD45.1^-^ CD45.2^+^ cell population among CD45^+^ cells in WT recipient mice, and the red bar represents the percentages of CD45.1^+^ CD45.2^-^ cell population in ESAM-KO recipient mice at stable state (5-FU day 0). (C-E) The number of BM cells from a pair of femora and tibiae was analyzed after 5-FU treatment (day 0 and day 8). The number of total BM MNC, CD3^+^ T cells, CD19^+^ B cells, MAC1^+^ myeloid cells (C), LSK cells (D), and c-Kit^-^ Ter119^+^ CD71^Hi^ erythroid progenitors (E) is shown. (F) PB Hb levels in 5-FU-treated WT and ESAM-KO mice recipients at day 8 are shown. (G, H) Equal amounts (2 × 10^5^) of BM cells from CD45.1 WT and CD45.2 ESAM-KO mice were mixed and transplanted to lethally irradiated CD45.1 WT mice (n = 6). Sixteen weeks after transplantation, half of them were sacrificed at 5-FU day 0, and others were treated with 150 mg/kg of 5-FU and sacrificed at day 8. (G) A scheme of the transplantation protocol is shown. (H) The percentages of CD45.1^-^ CD45.2^+^ cell population among the LSK fraction in the BM at 5-FU day 0 and day 8 are shown. Data are shown as mean ± SEM. Statistically significant differences are represented by an asterisk (*P < 0.05).

We previously observed that the recovery of the HSC-enriched LSK fraction after 5-FU treatment is delayed in ESAM-KO mice compared to WT mice [7]. Additionally, ESAM-KO mice experienced severe anemia after 5-FU treatment. Because ESAM is expressed in both endothelial and hematopoietic cells, it was unclear in which cells ESAM expression is essential for normal hematopoietic recovery. To address this issue, we made chimeric mice in our hematopoietic system by transplanting CD45.1 WT BM cells to CD45.2 ESAM-KO mice and CD45.2 WT BM cells to CD45.1 WT mice (Fig 4A). In the ESAM-KO mice recipients transplanted with WT BM cells, ESAM expression was absent in hematopoietic environmental cells, but present in hematopoietic cells. At 16 weeks after transplantation, these mice were analyzed in terms of the sensitivity of their hematopoietic system to 5-FU. Prior to 5-FU treatment, both WT and ESAM-KO recipient mice showed greater than 90% chimerism with transplanted cells in the PB (Fig 4B). The number of total BM MNC, CD3^+^ T cells, CD19^+^ B cells, MAC1^+^ myeloid cells, and LSK cells was not significantly different between 5-FU-treated WT and ESAM-KO mice at day 0 and day 8 (Fig 4C and 4D). Furthermore, no obvious reduction was observed in the 5-FU-treated ESAM-KO mice recipients regarding the number of c-Kit^-^ Ter119^+^ CD71^Hi^ mature erythroid progenitors or PB Hb levels at day 8 (Fig 4E and 4F). These results suggested that ESAM deficiency in the hematopoietic environment was not deleterious to the hematopoietic recovery.

Next, we made chimeric mice by mixing equal amounts of BM cells from untreated CD45.1 WT and CD45.2 ESAM-KO mice to then transplant them into CD45.1 WT recipients (Fig 4G). Sixteen weeks after transplantation, the contribution of ESAM-KO-derived hematopoietic cells from the BM was evaluated. ESAM-KO BM cells were able to robustly contribute to the hematopoietic reconstitution of lethally irradiated recipients (Fig 4H, 5-FU day 0). However, after 5-FU treatment, chimerism of ESAM-KO-derived cells in the LSK population significantly decreased, compared to that before the 5-FU treatment (Fig 4H, 5-FU day 8). These results suggested that ESAM-deficient hematopoietic cells are more sensitive to chemotherapy than WT cells, and that ESAM expression in hematopoietic cells is indispensable for normal hematopoietic recovery.

### ESAM Levels in Erythroid Progenitors Were Up-Regulated after BM Injury

We previously observed that 5-FU or irradiation treatment markedly up-regulates ESAM expression levels in LSK cells [7] (S5 Fig). Given that ESAM expression in hematopoietic cells is essential for normal hematopoietic recovery after a BM injury, up-regulation of ESAM might occur in a wide range of hematopoietic progenitors. Thus, we performed a detailed comparison of ESAM expression levels in hematopoietic progenitors before and after 5-FU treatment.

**Fig 5:**
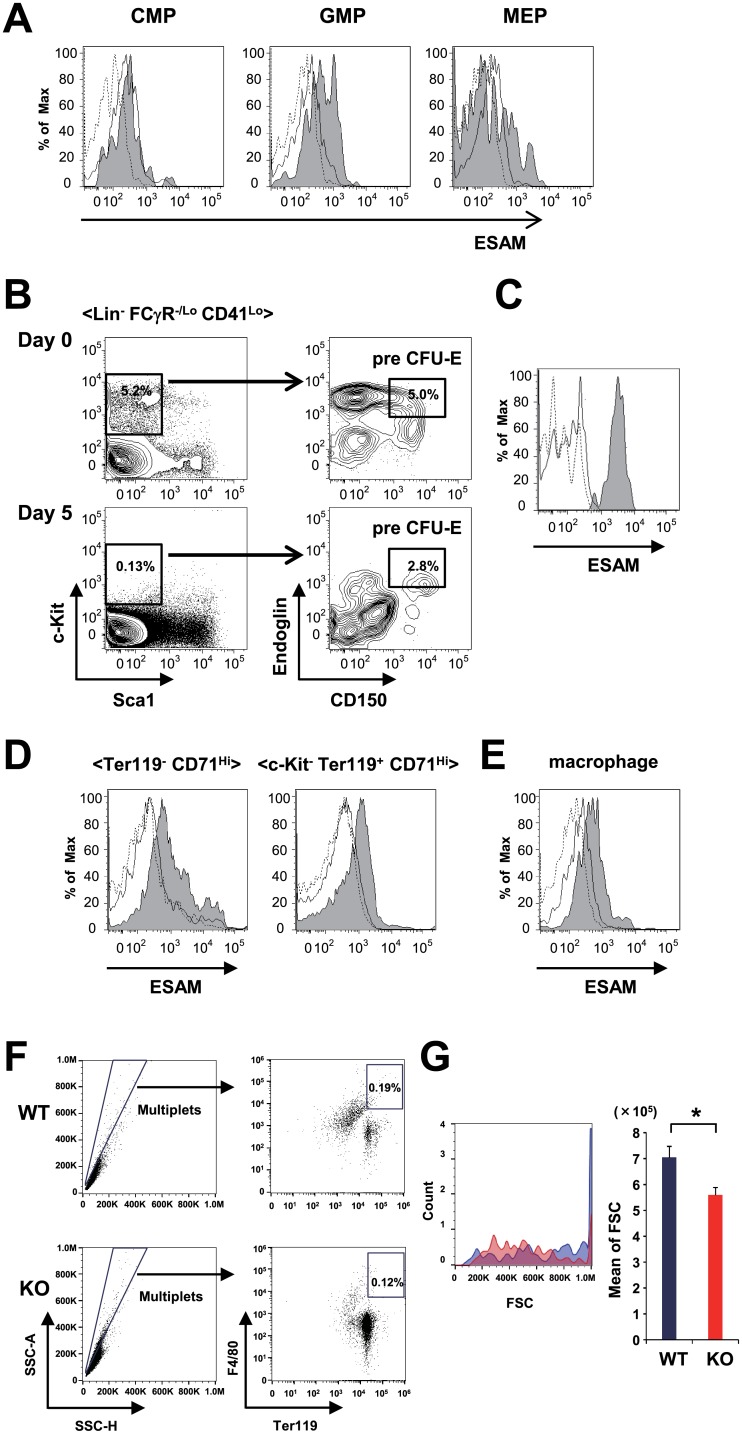
ESAM is up-regulated after 5-FU treatment in erythroid progenitors and macrophages. (A-E) C57BL/6J WT mice were treated with a single intravenous 5-FU (150 mg/kg) injection, and ESAM expression levels in myeloid or erythroid progenitors and macrophages in the BM were evaluated by FACS. In each histogram, the solid line and tinted line show ESAM levels after 5-FU treatment at day 0 (control) and day 5, respectively. The background level is added to each panel with an isotype control Ab (dashed line). (A) ESAM expression levels of CMP, GMP, and MEP fractions are shown. (B) Representative FACS profiles of pre CFU-E cells in BM after 5-FU treatment at day 0 and day 5 are shown. (C) ESAM expression levels in the pre CFU-E fraction are shown. (D) ESAM expression levels in the Ter119^-^ CD71^Hi^ fraction and the c-Kit^-^ Ter119^+^ CD71^Hi^ fraction are shown. (E) ESAM expression levels of Gr1^-^ F4/80^+^ CD115^Int^ SSC^Int/Lo^ macrophages are shown. (F) Representative FACS profiles of BM cells from 5-FU-treated WT and ESAM-KO mice (150 mg/kg) at day 8 are shown. Each number indicates the percentage of F4/80^+^ Ter119^+^ multiplets (erythroblastic islands). (G) In the left histogram, cell sizes determined by FSC in the erythroblastic island population of WT (blue) and ESAM-KO (red) mice are shown. In the right graph, the actual value of FSC is shown. Data are shown as mean ± SEM. Statistically significant differences are represented by an asterisk (*P < 0.05).

While ESAM expression was virtually negative in CMPs, GMPs, or MEPs before 5-FU treatment, expression levels in these progenitor cells were up-regulated after treatment (Fig 5A). Because the histogram of ESAM expression showed bimodal peaks in the MEP fraction after 5-FU, we separated the MEPs into ESAM^-^ and ESAM^+^ subpopulations, and tested their differentiation potential. Methylcellulose colony assays showed that ESAM^+^ MEPs exhibited higher colony-forming activity than ESAM^-^ MEPs. Interestingly, the ESAM^+^ MEP fraction contained a substantial number of primitive progenitor CFU-Mix (S6 Fig). The Lin^-^ FCγR^-/Lo^ CD41^Lo^ c-Kit^+^ Sca1^-^ endoglin^+^ CD150^+^ cell population, called pre CFU-E, was reported to contain mostly BFU-E and CFU-E (Fig 5B and [19]). Even though these cells do not express ESAM at the steady state, ESAM is markedly up-regulated by 5-FU treatment (Fig 5C). In Ter119^-^ CD71^Hi^ and c-Kit^-^ Ter119^+^ CD71^Hi^ erythroid progenitors, ESAM was also up-regulated (Fig 5D). Because erythroid progenitors from all the stages described above were damaged in the 5-FU-treated ESAM-KO mice (Fig 3), ESAM expression in those erythroid progenitors might play important roles in acute erythropoiesis.

BM macrophages can be transplanted by standard BM transplantation methods and are candidates for the erythroid progenitor niche [3, 20]. Therefore, we analyzed ESAM expression levels in Gr1^-^ F4/80^+^ CD115^Int^ SSC^Int/Lo^ macrophages before and after 5-FU treatment [21]. Intriguingly, although most of those BM macrophages were ESAM-negative at the steady state, a substantial number of them became ESAM-positive after 5-FU treatment (Fig 5E). The number of erythroblastic islands that are in Ter119^+^ F4/80^+^ multiplets are not significantly different between WT and ESAM-KO mice (data not shown) [3]. However, FACS profiles showed that forward scatter (FSC) of erythroblastic islands in WT mice were significantly higher than those in ESAM-KO mice, suggesting that the mean size of erythroblastic islands of WT mice was larger than that of ESAM-KO mice (Fig 5F and 5G). These results suggested that the up-regulation of ESAM might be functionally involved in the interaction between erythroid progenitors and their niche components during stress-induced acute erythropoiesis.

### Various Signaling Pathways Are Inhibited in ESAM-Deficient Erythroid Progenitors after 5-FU Treatment

**Fig 6:**
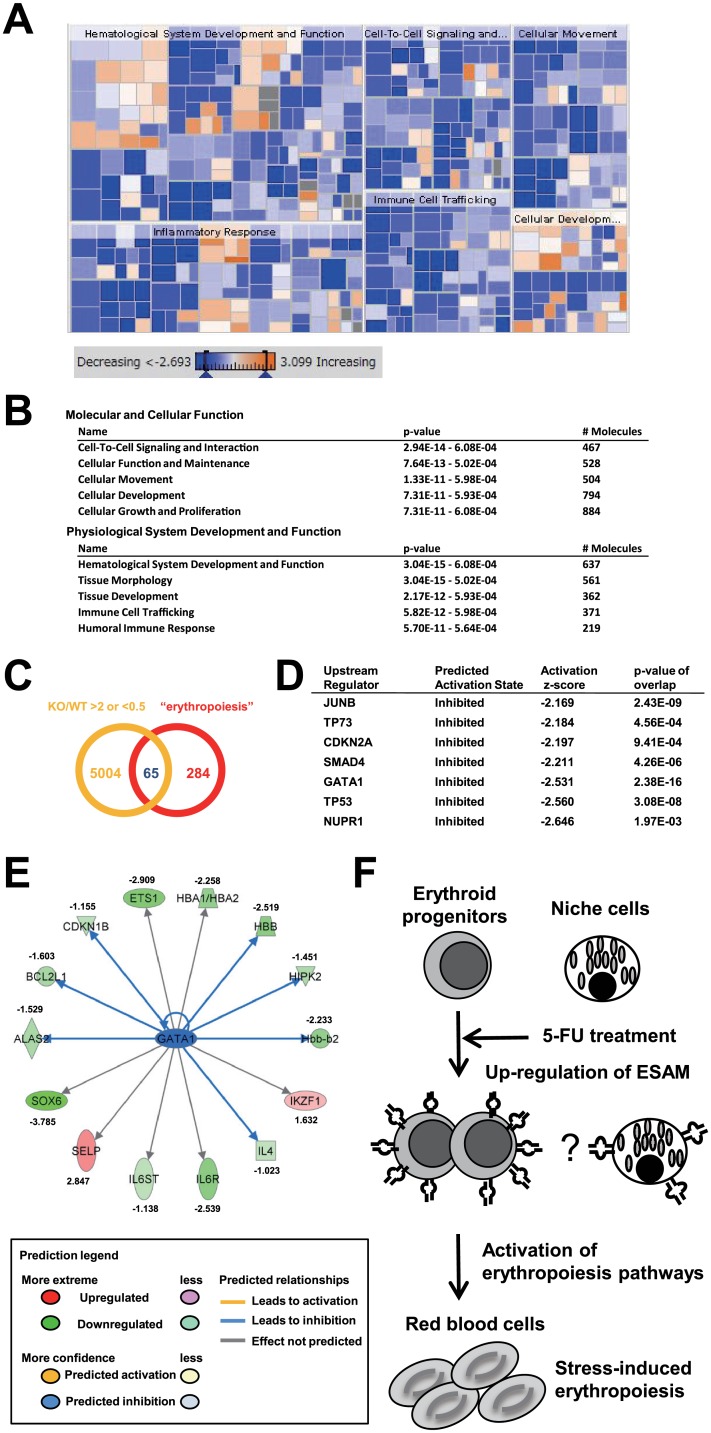
Differential gene expression in WT and ESAM-KO pre CFU-E fraction after 5-FU treatment. (A-E) WT and ESAM-KO mice were treated with a single 5-FU (120 mg/kg) injection and sacrificed 8 days after treatment. BM cells from each of the three mice were pooled and the cells in the pre CFU-E fraction were sorted. The extracted RNA samples were used to conduct microarray and bioinformatic analyses. Gene expression profiles of ESAM-KO pre CFU-E relative to its WT counterpart were evaluated. (A) As a result, a color-coded heat map analysis was obtained. This heat map allows for the visualization of the differential expression data of genes categorized by their functions using the Ingenuity Knowledge Base. The color bar indicates the *z*-score for each category: the strongest predicted increase (orange square) corresponds to *z*-score *>* 2, the strongest predicted decrease (blue square) corresponds to *z*-score *< –*2. Gray and white colors indicate categories with a –2 *< z*-score *<* 2 and without *z*-score, respectively. Larger squares indicate a more significant overlap among the genes altered in the dataset. In the heat map, the results of “hematological system development and function,” “inflammatory response”, “cell-to-cell signaling and interaction”, “immune cell trafficking”, “cellular movement”, and “cellular development” are shown. (B) The lists of differential expression data of genes categorized by their functions using the Ingenuity Knowledge Base. (C) Venn diagram of the 65 genes that are shared between the genes that have more than a 2-fold increased or decreased expression in ESAM-KO pre CFU-E compared to WT pre CFU-E, and the “erythropoiesis”-related genes. (D) The upstream regulator analyses were performed with respect to extracted genes in Fig 6C. The significantly altered upstream regulators are shown. (E) Genes downstream of *Gata1* are shown. Genes shown with green graphics are genes that are down-regulated, and those shown with red graphics are genes that are up-regulated in ESAM-KO mice. (F) A model of stress-induced erythropoiesis after 5-FU treatment is shown.

To determine the molecular mechanisms involved in the compromised erythropoiesis of ESAM-KO mice, Lin^-^ FCγR^-/Lo^ CD41^Lo^ c-Kit^+^ Sca1^-^ endoglin^+^ CD150^+^ cells were sorted from 5-FU-treated WT and ESAM-KO mice, and subjected to microarray analyses. Bioinformatic analyses using the Ingenuity Pathway Analysis (IPA) software revealed that the ESAM deficiency affected various signaling pathways such as “hematological system development”, “cell-to-cell signaling and interaction,” and “cellular movement” (Fig 6A and 6B). We focused on 65 genes that had the following characteristics: genes that were significantly affected by ESAM-deficiency and erythropoiesis-related genes (Fig 6C). Then, out of these 65 genes, upstream regulator analyses were performed to identify transcriptional regulators that can explain the changes in diverse gene expression. As a result, seven significantly altered upstream regulators were determined (Fig 6D). Interestingly, those upstream regulators were all inhibited in the ESAM-KO-derived population. Among the identified regulators, GATA1 is known as a key transcription factor of erythropoiesis in immature stages [22, 23]. We then confirmed that some genes downstream of *Gata1* were down-regulated in ESAM-KO mice (Fig 6E). On the other hand, IKAROS family zinc finger 1 (*Ikzf1)* expression was up-regulated in ESAM-KO mice. A previous study revealed that IKZF1 is a negative regulator for erythrocyte formation via repression of GATA1 [24, 25]. These results suggested that ESAM up-regulation in erythroid progenitors after 5-FU treatment induces differentiation or proliferation of erythroid cells, in part, via the activation of GATA1 signaling pathway. The embryonic ectoderm development (*Eed*) is a subunit of the polycomb repressive complex 2. Furthermore, Xie reported that Eed-KO BM HSCs are defective in differentiation [26]. We found marked similarities between the gene expression profiles of ESAM-KO pre CFU-E and Eed-KO HSCs (S7 Fig) [26]. In summary, we conclude that the up-regulation of ESAM in early erythroid progenitors is necessary to trigger activation of various signaling pathways that are needed for stress-induced erythropoiesis (Fig 6F).

## Discussion

While hematopoietic progenitors maintain a continuous cellular turnover of mature blood cells under a homeostatic state, they start to proliferate more actively after marrow ablation. Proliferation of early erythroid progenitors is also observed after acute anemia resulting from bleeding or hemolysis [15, 17, 27]. Prompt erythroid recovery after a BM injury is essential to maintain sufficient oxygen delivery to tissues. In clinical practice, delayed erythroid recovery after chemotherapy or irradiation in patients necessitates frequent red blood cell transfusions, often leading to iron overload and the risk of life-threatening complications. Therefore, understanding the mechanisms through which erythroid progenitors compensate for acute anemia is very important. In the present study, we have demonstrated that ESAM deficiency delays erythroid recovery after 5-FU administration by damaging a broad range of erythroid progenitors. Furthermore, ESAM is up-regulated in various types of hematopoietic cells in the BM, which are likely involved in the activation of signaling and transcriptional pathways that are critical for regulating erythropoiesis.

Adult ESAM-KO mice are slightly anemic, but otherwise healthy under a homeostatic state; however, they experienced severe pancytopenia after 5-FU treatment (Fig 1A and [7]). PB analyses, flow cytometry, and colony forming assays for the BM and spleen cells revealed that ESAM expression is indispensable for the immediate recovery of erythroid progenitors after BM injury. Furthermore, we found that ESAM expression is inducible after 5-FU treatment in erythroid progenitors at various differentiation stages, even though under a homeostatic state those progenitors scarcely bear this surface molecule (Fig 5A–5D). We previously reported that ESAM expression in HSCs was remarkably increased after a 5-FU injection, reaching its maximum peak on day 5 [7]. ESAM was also significantly up-regulated in early erythroid progenitors during days 5–7 after a 5-FU injection (Fig 5C and data not shown). Therefore, an up-regulation of ESAM in hematopoietic stem/progenitor cells after myelo-suppression is considered vital for early erythroid recovery.

Transplantation analyses suggested that ESAM expression in hematopoietic cells, but not in the BM environment, is essential for erythroid recovery. Furthermore, when sorted MEPs from 5-FU-treated BM were subjected to colony assays, ESAM-KO mice showed reduced BFU-E formation activity compared to WT mice. Therefore, it is important to consider how the up-regulation of ESAM in hematopoietic cells contributes to erythroid recovery (Fig 6F). One possibility is that the up-regulated ESAM in hematopoietic stem/progenitor cells directly transduces signals that promote erythroid proliferation and differentiation. ESAM has a proline-rich domain in the cytoplasmic region, which can mediate the interaction with signaling proteins containing SRC Homology 3 domains [28]. Indeed, the cytoplasmic domain of ESAM is known to interact with membrane-associated guanylate kinase to activate RhoA [29]. Another possibility is that progenitor cells that exhibit ESAM up-regulation might be able to interact with their niche components more intimately and receive appropriate signals for erythropoiesis. A previous study showed that ESAM-overexpressing CHO cells tend to form cell aggregates, likely via homophilic interactions of ESAM proteins [28]. In this context, it is noteworthy that CD169^+^ macrophages in the BM also become ESAM-positive after 5-FU treatment because these macrophages are known to be critical for erythroid recovery from acute anemia or myeloablation [3]. Additionally, because erythroblastic islands of ESAM-KO mice were reduced in size (when compared with WT mice), ESAM might correlate with the interactions between erythroblasts and macrophages.

Our microarray analyses for the pre CFU-E fraction suggested that the expression levels of many genes related to “hematological system development and function”, “inflammatory response”, “cell-to-cell signaling and interaction”, “immune cell trafficking”, “cellular movement”, and “cellular development”, were decreased in the ESAM-KO mice (Fig 6A and 6B). Interestingly, all of the regulators identified by pathway analyses were inhibited in the ESAM-KO pre CFU-E (Fig 6D). GATA1 is a key transcription factor that activates various erythroid-specific genes during erythroid maturation [30]. Downstream of *Gata1*, some genes including hemoglobin alpha chain complex (*Hba*) and hemoglobin beta chain complex (*Hbb*), which encode globin proteins, were down-regulated in ESAM-KO mice, suggesting that ESAM deficiency negatively affects Hb synthesis after BM injury. GATA1 is also required for the differentiation of the BFU-E stage to the CFU-E stage [31]. Thus, it is noteworthy that the number of CFU-E and c-Kit^-^ Ter119^+^ CD71^Hi^ mature erythroid progenitors was significantly lower in 5-FU-treated ESAM-KO mice compared to that in 5-FU-treated WT mice (Fig 3B and 3D).

Whereas *JunB*, *Tp73*, *Cdkn2a*, *Smad4*, *Tp53*, and *Nupr1* are known cell-cycle related genes, *JunB*, *Tp73*, *Cdkn2a*, and *Nupr1* are also reported to induce erythropoiesis [32–35]. JUNB, a member of the activator protein-1 family of transcription factors, induces erythroid differentiation with a slower rate of proliferation [33]. Tap73, a protein isoform from the *Tp73* gene, directly induces the transcription of the *Gata1* gene [34]. CDKN2A (p16), a member of the INK4 family, induces cell-cycle arrest or apoptosis, and promotes erythroid differentiation in erythroid lineage cells [35]. EPO induces the cell-cycle progression factor NUPR1 [32]. The roles of TP53 are not unidirectional in terms of erythroid differentiation. Artificial expression of TP53 induces erythroid differentiation in chronic myelogenous leukemia cells; it also induces cell-cycle arrest and anemia in disorders such as Diamond-Blackfan anemia and the 5q^-^ syndrome [36, 37]. In addition to the GATA1 pathway, our microarray data identified those critical regulators for erythropoiesis as the down-stream molecules of ESAM. The similarities in the gene expression profiles of ESAM-KO and Eed-KO mice strengthen the conclusion that ESAM is a possible essential player in diverse pathways regulating erythroid differentiation and proliferation.

In summary, our data have shown that ESAM expression in hematopoietic cells plays essential roles in stress-induced erythropoiesis after a BM injury. Additionally, the up-regulation of ESAM in primitive erythroid progenitors is necessary for activation of differentiation- or proliferation-related genes that contribute to erythropoiesis. We are now investigating the expression pattern and function of the human ESAM in HSPCs (T. Ishibashi et al. manuscript in preparation). Additional research on the human ESAM will lay the foundation for clinical applications. Furthermore, in the field of regenerative medicine, ESAM-positive cells in combination with other specific markers will be useful to purify active erythroid progenitor cells.

## Supporting Information

S1 Fig Erythroblast subsets in steady state BM cells and splenocytes from WT and ESAM-KO mice. BM cells (A-B) and splenocytes (C-D) were isolated from WT and ESAM-KO mice to perform FACS analyses. (A, C) Representative FACS profile of BM cells (A) and splenocytes (C) stained with antibodies against Ter119 and CD71. In the right panel, Ter119^Hi^ cells were analyzed with respect to FSC. (B, D) In the left graph, the percentage of Ter119^Med^ CD71^Hi^ proerythroblasts (ProE.) in whole cells is shown. In the right graph, percentages of Ery.A (Ter119^Hi^ CD71^Hi^ FSC^Hi^), Ery.B (Ter119^Hi^ CD71^Hi^ FSC^Lo^), and Ery.C (Ter119^Hi^ CD71^Lo^ FSC^Lo^) erythroblast populations within Ter119^Hi^ cells are shown (BM; n = 5 in each, spleen; n = 4 in each). Data are shown as mean ± SEM. Statistically significant differences are represented by asterisks (*P < 0.05, ** *P* < 0.01).
(PDF)

S2 Fig Erythroblast subsets in 5-FU-treated BM cells and splenocytes from WT and ESAM-KO mice. WT and ESAM-KO mice were injected with 150 mg/kg 5-FU. Then, 8 days after treatment, BM cells (A-B) and splenocytes (C-D) were isolated to perform FACS analyses. (A, C) Representative FACS profile of BM cells (A) and splenocytes (C) are shown. (B, D) In the left graph, the percentage of proerythroblasts (ProE.) in whole cells is shown. In the right graph, percentages of Ery.A, Ery.B, and Ery.C erythroblast populations within Ter119^Hi^ cells are shown (BM; n = 5 in each, spleen; n = 6 in each). Data are shown as means ± SEM. Statistically significant differences are represented by asterisks (*P < 0.05, ** *P* < 0.01).
(PDF)

S3 Fig ESAM is unessential for erythroid proliferation after hemolytic anemia induced by PHZ administration. WT and ESAM-KO mice were treated with 40 mg/kg of PHZ intraperitoneally for 2 consecutive days (days 0 and 1) to then sacrifice the mice and analyze erythroid recovery at day 6 (n = 3 in each). (A) Hemoglobin concentration in PB is shown. (B) 1 × 10^5^ BM cells or splenocytes were plated for counting BFU-E. Each bar represents the number of BFU-E in BM (left graph) or spleen (right graph). (C) The number of c-Kit^-^ Ter119^+^ CD71^Hi^ cells in the BM (left graph) or spleen (right graph) are shown. Data are shown as mean ± SEM.
(PDF)

S4 Fig The vulnerability of erythropoiesis in ESAM-KO mice is not due to hemolysis or insufficient production of EPO, SCF, or BMP-4. (A-F) Comparison analyses between WT and ESAM-KO mice treated with 150 mg/kg of 5-FU were performed. Serum total bilirubin (T-bil) (A) and serum erythropoietin (EPO) (B) were evaluated (n = 3 in each). T-bil was analyzed using Vetscan VS2 and serum EPO was analyzed by BML, INC. (C) Pre CFU-E progenitor cells were sorted from pooled BM cells (two mice in each), and gene expression levels of *EpoR* were evaluated. (D) Femurs were flushed with 0.3 mL of PBS. Cells were lysed by a freeze and thaw cycle. Cell debris was removed by centrifugation. Then, the concentration of SCF in the BM was analyzed using a mouse SCF ELISA kit. (E-F) Expression levels of *SCF* (E) and *Bmp-4* (F) in BM cells and splenocytes are shown. (C, E-F) RNA samples were isolated using a PureLink RNA Mini Kit. Reverse transcription reactions were performed using a High Capacity RNA-to-cDNA Kit. Relative expression of each gene relative to GAPDH were evaluated according to the Taqman Gene Expression Assay Protocol. (A-B, D-F) Data are shown as mean ± SEM. Statistically significant differences are represented by an asterisk (*P < 0.05).
(PDF)

S5 Fig ESAM is up-regulated on HSPCs after total body irradiation. ESAM expression levels on BM LSK fractions from C57BL/6 WT mice after 5.5Gy total body irradiation (TBI) were examined using a FACS analysis. Each panel shows a representative histogram of ESAM expression level on LSK at day 0 (control), 4, and 8 after TBI. Dashed lines show background levels with an isotype control Ab. Tinted lines show ESAM expression levels of LSK after TBI. The solid line, which represents ESAM expression levels at day 0 is added to each panel. Upper and lower numbers in each histogram indicate the percentages of ESAM^+^ and ESAM^Hi^ cells, respectively.
(PDF)

S6 Fig ESAM^+^ MEP fraction contains a substantial number of primitive progenitor CFU-Mix. ESAM^-^ or ESAM^+^ MEPs were sorted from C57BL/6 WT mice under steady state or 8 days after 150 mg/kg of 5-FU injection. Then 1,500 cells were plated in methylcellulose media, Methocult GF M 3434. Under the steady state, an ESAM^+^ MEP population could not be detected. After 10 days, CFU-Mix colonies were enumerated according to shape and color under an inverted microscope (n = 3 in each). N.D. means “not done”. Data are shown as mean ± SEM. Statistically significant differences are represented by an asterisk (** *P* < 0.01).
(PDF)

S7 Fig Changes in gene expression resulting from ESAM deficiency correlates with those resulting from Eed deficiency. Genes that were differently expressed between WT and ESAM-KO pre CFU-E (Bioset 1) were compared with those in WT and Eed-KO HSCs (Bioset 2) using the NextBio software. The Bioset 2 was extracted from the GEO experiment accession number GSE 51084. (A) Venn diagram shows the number of common and unique genes in both sets. (B) Significance of the overlap between 2 gene subsets. The scale bar indicates–log (p-value). The comparison shows a positive correlation between these 2 biosets.
(PDF)
