# Supplementary figures and images for: Endothelial Cell-Selective Adhesion Molecule Expression in Hematopoietic Stem/Progenitor Cells Is Essential for Erythropoiesis Recovery after Bone Marrow Injury
Source: PLoS One. 2016 Apr 25;11(4):e0154189. doi: 10.1371/journal.pone.0154189 (PMC4844162; doi:10.1371/journal.pone.0154189)

S1 Fig

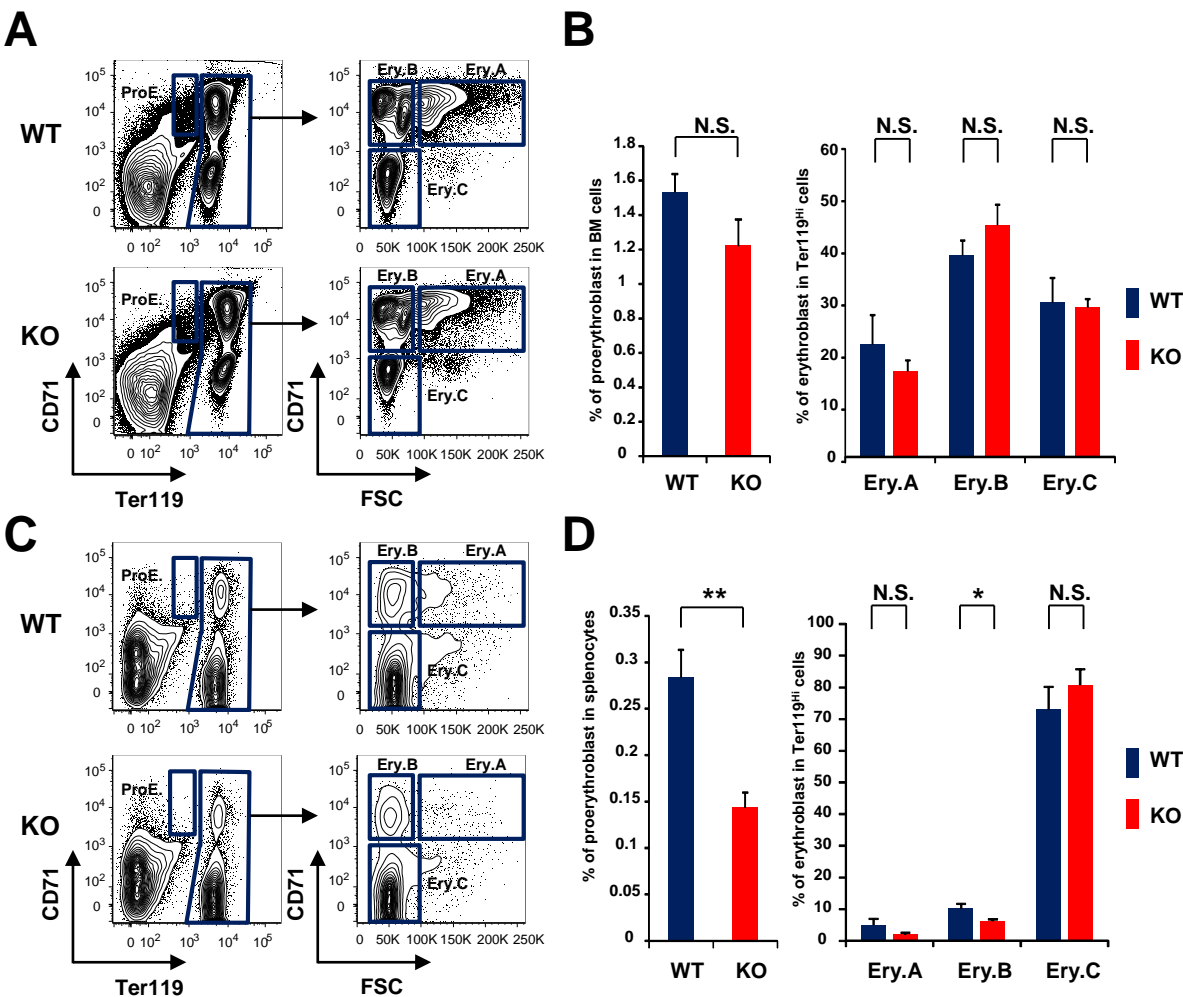

Supplement: S1 Fig — BM cells (A-B) and splenocytes (C-D) were isolated from WT and ESAM-KO mice to perform FACS analyses. (A, C) Representative FACS profile of BM cells (A) and splenocytes (C) stained with antibodies against Ter119 and CD71. In the right panel, Ter119Hi cells were analyzed with respect to FSC. (B, D) In the left graph, the percentage of Ter119Med CD71Hi proerythroblasts (ProE.) in whole cells is shown. In the right graph, percentages of Ery.A (Ter119Hi CD71Hi FSCHi), Ery.B (Ter119Hi CD71Hi FSCLo), and Ery.C (Ter119Hi CD71Lo FSCLo) erythroblast populations within Ter119Hi cells are shown (BM; n = 5 in each, spleen; n = 4 in each). Data are shown as mean ± SEM. Statistically significant differences are represented by asterisks (*P < 0.05, ** P < 0.01). (PDF) [file pone.0154189.s001.pdf]

S2 Fig

**A**

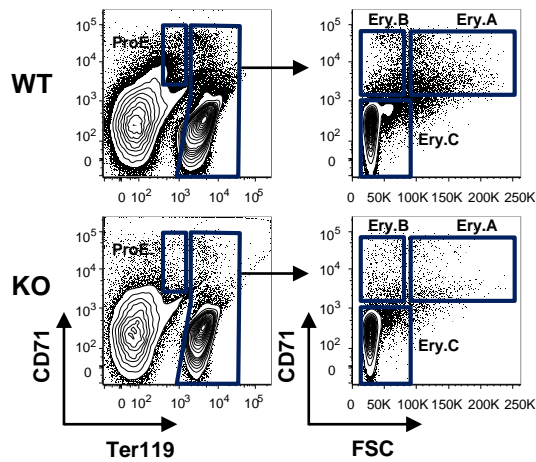

**B**

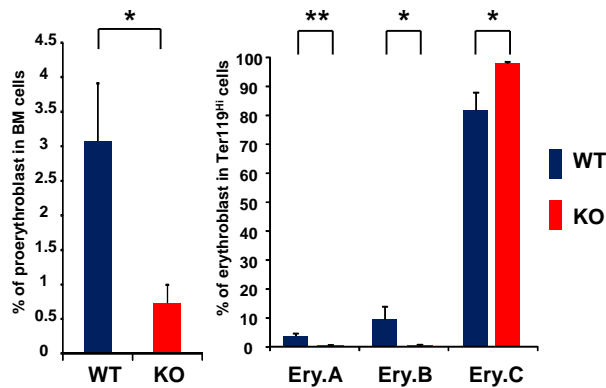

**C**

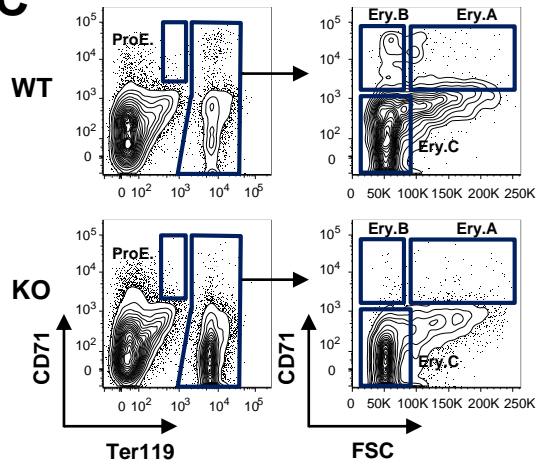

**D**

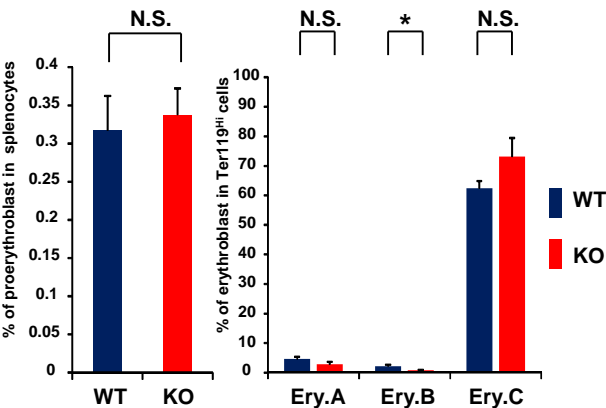

Supplement: S2 Fig — WT and ESAM-KO mice were injected with 150 mg/kg 5-FU. Then, 8 days after treatment, BM cells (A-B) and splenocytes (C-D) were isolated to perform FACS analyses. (A, C) Representative FACS profile of BM cells (A) and splenocytes (C) are shown. (B, D) In the left graph, the percentage of proerythroblasts (ProE.) in whole cells is shown. In the right graph, percentages of Ery.A, Ery.B, and Ery.C erythroblast populations within Ter119Hi cells are shown (BM; n = 5 in each, spleen; n = 6 in each). Data are shown as means ± SEM. Statistically significant differences are represented by asterisks (*P < 0.05, ** P < 0.01). (PDF) [file pone.0154189.s002.pdf]

S3 Fig

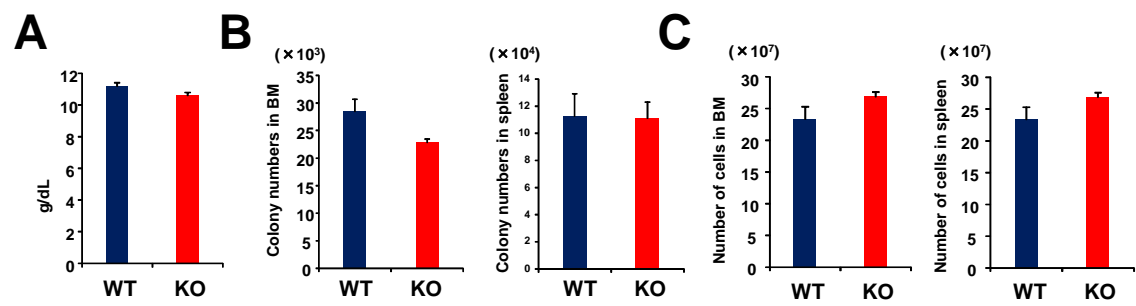

Supplement: S3 Fig — WT and ESAM-KO mice were treated with 40 mg/kg of PHZ intraperitoneally for 2 consecutive days (days 0 and 1) to then sacrifice the mice and analyze erythroid recovery at day 6 (n = 3 in each). (A) Hemoglobin concentration in PB is shown. (B) 1 × 10^5^ BM cells or splenocytes were plated for counting BFU-E. Each bar represents the number of BFU-E in BM (left graph) or spleen (right graph). (C) The number of c-Kit- Ter119+ CD71Hi cells in the BM (left graph) or spleen (right graph) are shown. Data are shown as mean ± SEM. (PDF) [file pone.0154189.s003.pdf]

S4 Fig

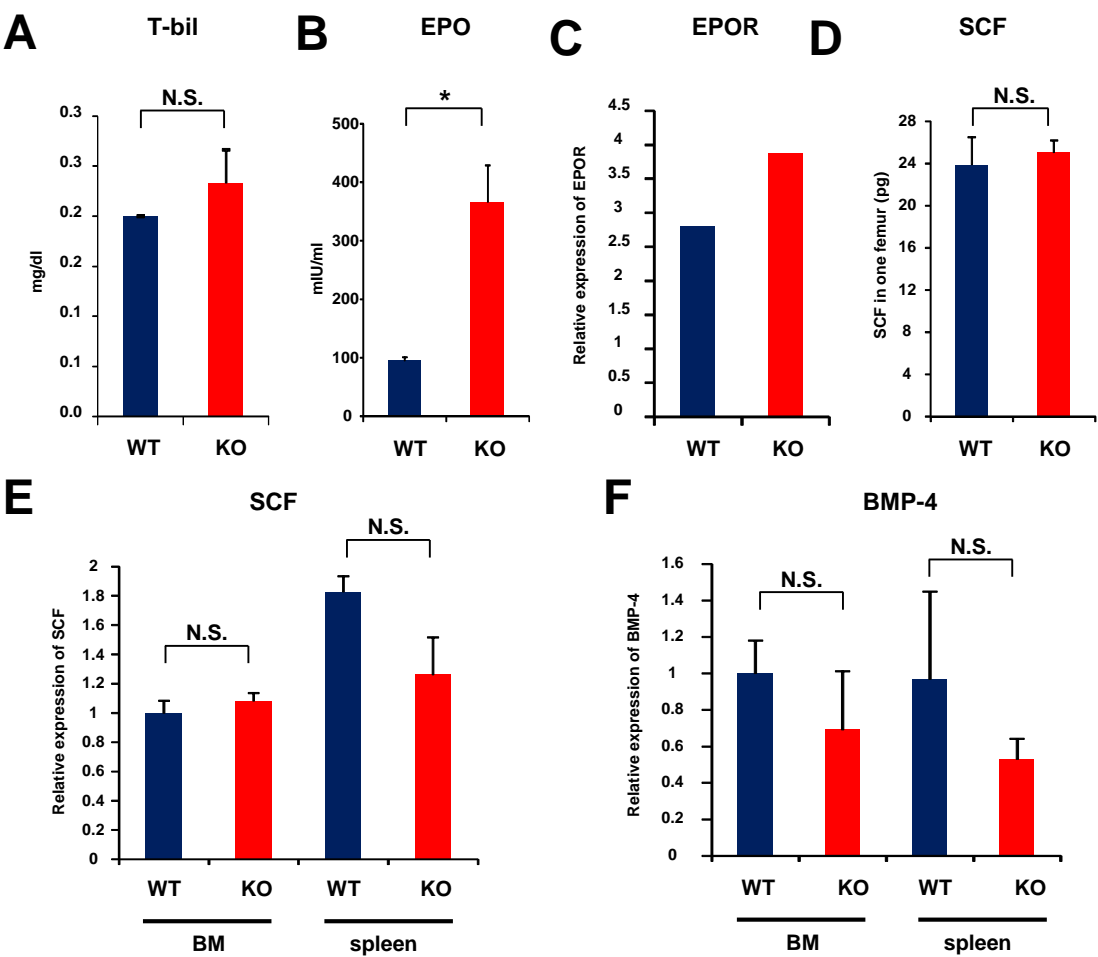

Supplement: S4 Fig — (A-F) Comparison analyses between WT and ESAM-KO mice treated with 150 mg/kg of 5-FU were performed. Serum total bilirubin (T-bil) (A) and serum erythropoietin (EPO) (B) were evaluated (n = 3 in each). T-bil was analyzed using Vetscan VS2 and serum EPO was analyzed by BML, INC. (C) Pre CFU-E progenitor cells were sorted from pooled BM cells (two mice in each), and gene expression levels of EpoR were evaluated. (D) Femurs were flushed with 0.3 mL of PBS. Cells were lysed by a freeze and thaw cycle. Cell debris was removed by centrifugation. Then, the concentration of SCF in the BM was analyzed using a mouse SCF ELISA kit. (E-F) Expression levels of SCF (E) and Bmp-4 (F) in BM cells and splenocytes are shown. (C, E-F) RNA samples were isolated using a PureLink RNA Mini Kit. Reverse transcription reactions were performed using a High Capacity RNA-to-cDNA Kit. Relative expression of each gene relative to GAPDH were evaluated according to the Taqman Gene Expression Assay Protocol. (A-B, D-F) Data are shown as mean ± SEM. Statistically significant differences are represented by an asterisk (*P < 0.05). (PDF) [file pone.0154189.s004.pdf]

S5 Fig

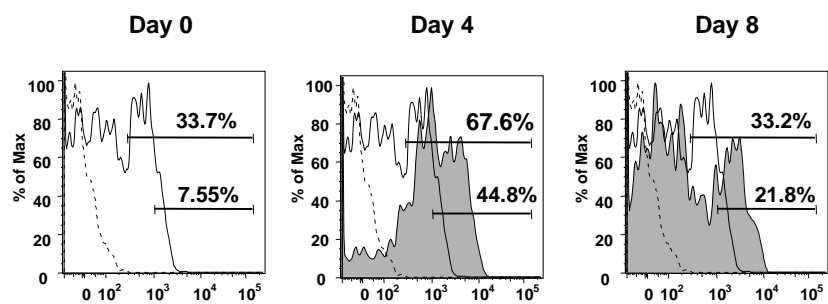

Supplement: S5 Fig — ESAM expression levels on BM LSK fractions from C57BL/6 WT mice after 5.5Gy total body irradiation (TBI) were examined using a FACS analysis. Each panel shows a representative histogram of ESAM expression level on LSK at day 0 (control), 4, and 8 after TBI. Dashed lines show background levels with an isotype control Ab. Tinted lines show ESAM expression levels of LSK after TBI. The solid line, which represents ESAM expression levels at day 0 is added to each panel. Upper and lower numbers in each histogram indicate the percentages of ESAM+ and ESAMHi cells, respectively. (PDF) [file pone.0154189.s005.pdf]

S6 Fig

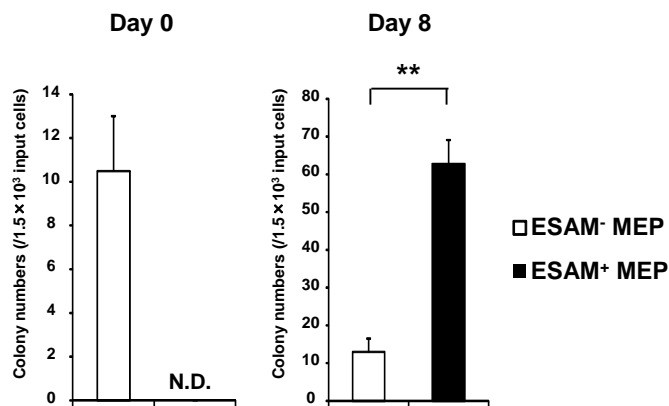

Supplement: S6 Fig — ESAM- or ESAM+ MEPs were sorted from C57BL/6 WT mice under steady state or 8 days after 150 mg/kg of 5-FU injection. Then 1,500 cells were plated in methylcellulose media, Methocult GF M 3434. Under the steady state, an ESAM+ MEP population could not be detected. After 10 days, CFU-Mix colonies were enumerated according to shape and color under an inverted microscope (n = 3 in each). N.D. means “not done”. Data are shown as mean ± SEM. Statistically significant differences are represented by an asterisk (** P < 0.01). (PDF) [file pone.0154189.s006.pdf]

S7 Fig

A

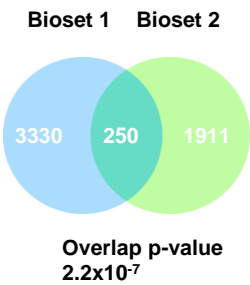

B

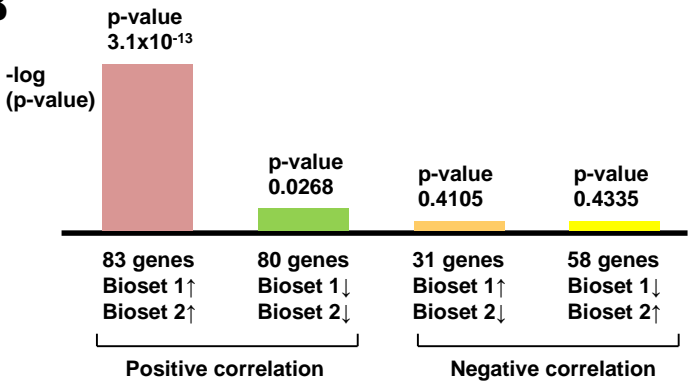

Supplement: S7 Fig — Genes that were differently expressed between WT and ESAM-KO pre CFU-E (Bioset 1) were compared with those in WT and Eed-KO HSCs (Bioset 2) using the NextBio software. The Bioset 2 was extracted from the GEO experiment accession number GSE 51084. (A) Venn diagram shows the number of common and unique genes in both sets. (B) Significance of the overlap between 2 gene subsets. The scale bar indicates–log (p-value). The comparison shows a positive correlation between these 2 biosets. (PDF) [file pone.0154189.s007.pdf]
